# Factors associated with exclusive breastfeeding during postpartum in Lanzhou city, China: a cross-sectional study

**DOI:** 10.3389/fpubh.2023.1089764

**Published:** 2023-08-30

**Authors:** Yuelu Chen, Yong Zhao, Wenling Wang, Fengdi Wang, Huimin Jiang, Lianlian Wang

**Affiliations:** ^1^Department of Reproductive Health and Infertility, First Affiliated Hospital of Chongqing Medical University, Chongqing, China; ^2^Chongqing Key Laboratory of Maternal and Fetal Medicine, Chongqing Medical University, Chongqing, China; ^3^College of Public Health and Management, Chongqing Medical University, Chongqing, China; ^4^Perinatology Center, Gansu Provincial Maternity and Child-Care Hospital, Lanzhou, Gansu, China

**Keywords:** exclusive breastfeeding, risk factors, breastfeeding knowledge, China, postpartum

## Abstract

**Aim:**

Breastfeeding generates short-term and long-term benefits for both mother and child. Exclusive breastfeeding (EBF) is promoted in China for years, but its practice still lags far behind the international average, even among low- and middle- income countries. This study aimed to investigate factors associated with EBF during postpartum.

**Methods:**

This study was conducted in a tertiary referral hospital in Gansu Province, Northwest China from October 2019 to April 2020. 3,738 postnatal women were finally included and each of them completed an elaborately designed questionnaire. Infant feeding patterns (EBF or not) and reasons for NEBF (non-exclusive breastfeeding) were collected. The feeding knowledge score was based on 17 questions in relation to breastfeeding. The total score ranges from 0 to 17. Higher score means better understanding about breastfeeding knowledge. Multivariate logistic regression models were used to determine associated factors of EBF during postpartum. A subgroup analysis was conducted to investigate the association between feeding knowledge score and exclusive breastfeeding.

**Results:**

Six weeks after childbirth, 1891 mothers (50.6%) maintained EBF. Among the NEBF mothers, 57.01% (*n* = 1,053) of them stopped exclusive breastfeeding due to self-perceived lack of breast milk production. Factors associated with NEBF were higher maternal age, ethnic minorities and cesarean section. Protective factors of EBF included multipara, positive feeding attitude and high breastfeeding knowledge score. In subgroup analysis, we found the breastfeeding knowledge score had a significant impact on the mothers of Han nationality, underwent cesarean or natural delivery, both primiparous and multiparous, and those with positive attitude towards breastfeeding (*p* < 0.05).

**Conclusion:**

We need a comprehensive and individualized framework of strategies to support children, mothers and their families. During puerperium, improving maternal knowledge of breastfeeding is beneficial to EBF practice. However, for ethnic minorities and those with less active breastfeeding attitudes, breastfeeding knowledge is of limited use, more researches are needed to explore the uncovered reasons, so that more personalized interventions could be developed for them.

## 1. Introduction

Breastfeeding is able to benefit both mother and child creating a win-win situation. For mothers, it helps to regain weight, reduce the risk of breast cancer, ovarian cancer and postpartum depression etc. For babies, 6 months of exclusive breastfeeding generates short-term and long-term benefits (1, 2), reducing the prevalence of respiratory diseases, diarrhea, cutting down the mortality of preterm infants, helping intellectual development, immunity construction, impacting their adaptability, and language ability in a positive way later in life (3).

Of 101 countries with valid data, 32 have reached the WHA (World Health Assembly) target, i.e., 6 months’ EBFR (Exclusive Breast-Feeding Rate) should achieve 50% by 2025 (4, 5). Despite of all the benefits, the EBFR in China is still unsatisfactory and lags far behind the international average. In low- or middle-income countries, about 37% of infants were breastfed exclusively (4, 6) while in China that number was barely 21% by 2013 (7). Even by 2019, China still suffers from a low EBFR for the first 6 month ranging from 20 to 36% in both urban and rural areas (4, 7). A previous study showed that the EBFR in China at the end of the first, third, and sixth month after childbirth were 81.94, 68.52 and 31.94%, respectively (8).

Based on previous studies in Southeast Asia and the Pacific Area (9–12), Middle East (13–15), Europe (16–18) and the North and South Americas (19–21), we found a wide range of factors at individual, cultural, health facility, and socioeconomic levels. However, the effects of some factors sometimes reached contradictory conclusions (2). Aside from that, a majority of the previous studies have examined the risk factors of exclusive breastfeeding focusing on 6 months after childbirth, few have yet focused on puerperium period. Notably, the puerperium is a critical point, which provides clinicians opportunity to intervene in a face-to-face way, since postpartum women visit the hospital for routine maternal checkups 6 weeks after delivery. However, few studies have focused on factors influencing exclusive breastfeeding during this period of time. To provide theoretical support to healthcare professionals, we surveyed 3,738 women who underwent postpartum checkups in the obstetrics department of Gansu Provincial Maternity and Childcare Hospital from October 2019 to April 2020, and used subgroup analysis to help healthcare professionals develop targeted and individualized interventions.

## 2. Methods

### 2.1. Study design

The cross-sectional survey was conducted at Gansu Provincial Maternity and Childcare Hospital in Lanzhou, Gansu Province, in Northwest China from October 2019 to April 2020.

### 2.2. Participants

The target population were puerperal women who visited the obstetrics department of this hospital on their sixth week after childbirth and met the following criteria (a) willing to participate, (b) aged over 19 and under 49, (c) capable of Chinese reading and writing, and (d) gave birth to a single child this time.

A total of 4,170 postnatal women completed an elaborately designed questionnaire, and according to the inclusion criteria, we excluded those who gave birth to twins or three children this time and those lack of valid data, 3,738 postnatal women were finally included. 1847 mothers adopted bottle feeding or mixed feeding were categorized into NEBF group (Non-exclusive breastfeeding), while 1891 mothers adopted exclusive breastfeeding were categorized into EBF group (Exclusive breastfeeding). This study was approved by the Ethics Committee of The First Affiliated Hospital of Chongqing Medical University on October 22, 2018 with approval number 2018-131. All participants in this study provided informed consent.

### 2.3. Sample size

The study was a cross-sectional survey and the outcome indicators were dichotomous outcomes, so we calculated the sample size by the formula *n* = *Z*_α_^2^ * *p* *(1 − *p*)/*δ*^2^. Previous literature lacks the rate of EBF at 42 days postpartum, a study showed that the EBFR in China at the end of the first, third month after childbirth were 81.94 and 68.52%, respectively, Duan et al. (7). We chose *p* = 0.685 to ensure an adequate sample size. For a confidence level of 95%, α is 0.05 and Z1 − α/2 = 1.96. The required sample size *N* = 2073. If we anticipate a 10 ~ 20% of the inefficiency, we may want to increase the sample size and total sample size *n* as 2,280 ~ 2,487.

### 2.4. Definition of terms

Nationality was categorized into Han and Minority while residence included urban and rural area. Educational level was divided into three degrees including low (junior high school and below), median (senior high school) and high (undergraduate, graduate and above). Delivery mode included natural vaginal delivery and cesarean section. Parity was either primipara or multipara. Moreover, we recoded family monthly income as low (<¥4,500), medium (¥4,500–9,000) and high (>¥9,000). Breast or nipple abnormalities included mastitis, breast engorgement, and cracked nipples. Feeding attitude reflected mothers’ subjective standpoints towards exclusive breastfeeding (willing/ neutral or unwilling).

Edinburgh Postnatal Depression Scale (EPDS) is a widely preformed self-report scale used among postnatal women 6 weeks after childbirth to screen Postnatal Depression. This scale consists of 10 items and the score of each item ranges from 0 to 4 describing women’s subjective feelings. The total score is ranging from 0 to 30, and ≥ 13 can be defined as PPD (Postpartum Depression).

The feeding knowledge score was based on 17 questions in relation to breastfeeding. Mothers were asked to read the questions such as “The sooner you start breastfeeding, the better” and identify true or false (gain 1 point for correct answer, and account 0 point for both no knowledge and wrong answer). The total score ranges from 0 to 17. Higher score means better understanding about breastfeeding knowledge (Breastfeeding knowledge content see Supplementary materials).

Five categories addressing the main reasons for cessation of EBF were developed based on previous studies. These were: (1) “Perceived low milk quantity” defined as mother’s self-reported perception that the infant was not getting enough milk and showing signs of hunger, (2) “Return to work” meant the mother returned to work or planned to do so, (3) “Worry about body shape” defined as mother’s decision to stop breastfeeding due to considering body shape, (4) “Mother’s medical condition” which included medical conditions related to breastfeeding as well as the advice of a doctor or healthcare professional, and (5) “Others” defined as EBF cessation with no further explanation.

### 2.5. Statistical analysis

Analyses were completed using SPSS version 25 (SPSS Inc., Chicago, IL, United States). Visualization in subgroup analysis were accomplished by R (version 3.6.3) and ggplot2 (version 3.3.3) R package. Frequency and percentage were used to describe the categorical variables while mean ± standard deviation was applied for continuous variables. The relationship between outcome variable and continuous, normally distributed independent variables (age, feeding knowledge score and BMI) were evaluated by *t*-test. Other categorical variables were correspondingly analyzed by Chi-square test. In order to investigate the possible associated factors of exclusive breastfeeding during puerperium, binary logistic regression was calculated. Variables with *p* < 0.1 were included in the regression model to control confounding. For the feeding knowledge score, we did subgroup analyses by age (<35 versus ≥35), nationality, delivery mode, parity and feeding attitude. In all statistical tests, a value of *p* < 0.05(double-sided) was considered significant.

## 3. Results

### 3.1. Socio-demographic characteristics of sample participants

A total of 3,738 participants were recruited, demographic, and clinical characteristics of them were described in Table 1. The mean age was 30.23 years (SD = 3.83). Up to 6 weeks after delivery, 50.6% of mothers maintained EBF. The majority of them were of Han nationality, achieved high educational level and lived in urban areas.

**Table 1:** Socio-demographic characteristics of sample participants.

Variables		Mean (SD) or *N* (%)
Total		3,738
Age		30.23 (3.83)
BMI		21.26 (3.00)
Nationality	Han	3,462 (92.6)
	Minority	276 (7.4)
Residence	Urban	3,394 (90.8)
	Rural	344 (9.2)
Educational level	Low	418 (11.2)
	Median	232 (6.2)
	High	3,088 (82.6)
Parity	Primipara	2,592 (69.3)
	Multipara	1,146 (30.7)
Delivery	Natural delivery	2,492 (66.7)
	Cesarean section	1,246 (33.3)
Feeding attitude	Neutral or unwilling	167 (4.47)
	Willing	3,574 (95.61)
Feeding mode	NEBF (Non-exclusive breastfeeding)	1847 (49.4)
	EBF (Exclusive breastfeeding)	1891 (50.6)

Chi-square test, independent *t*-test. *p < 0.05, **p < 0.01, ***p < 0.001. NEF, non-exclusive breast feeding (bottle feeding or mixed feeding); EF, exclusive breast feeding.

### 3.2. Reasons for breastfeeding cessation

**Figure 1:**
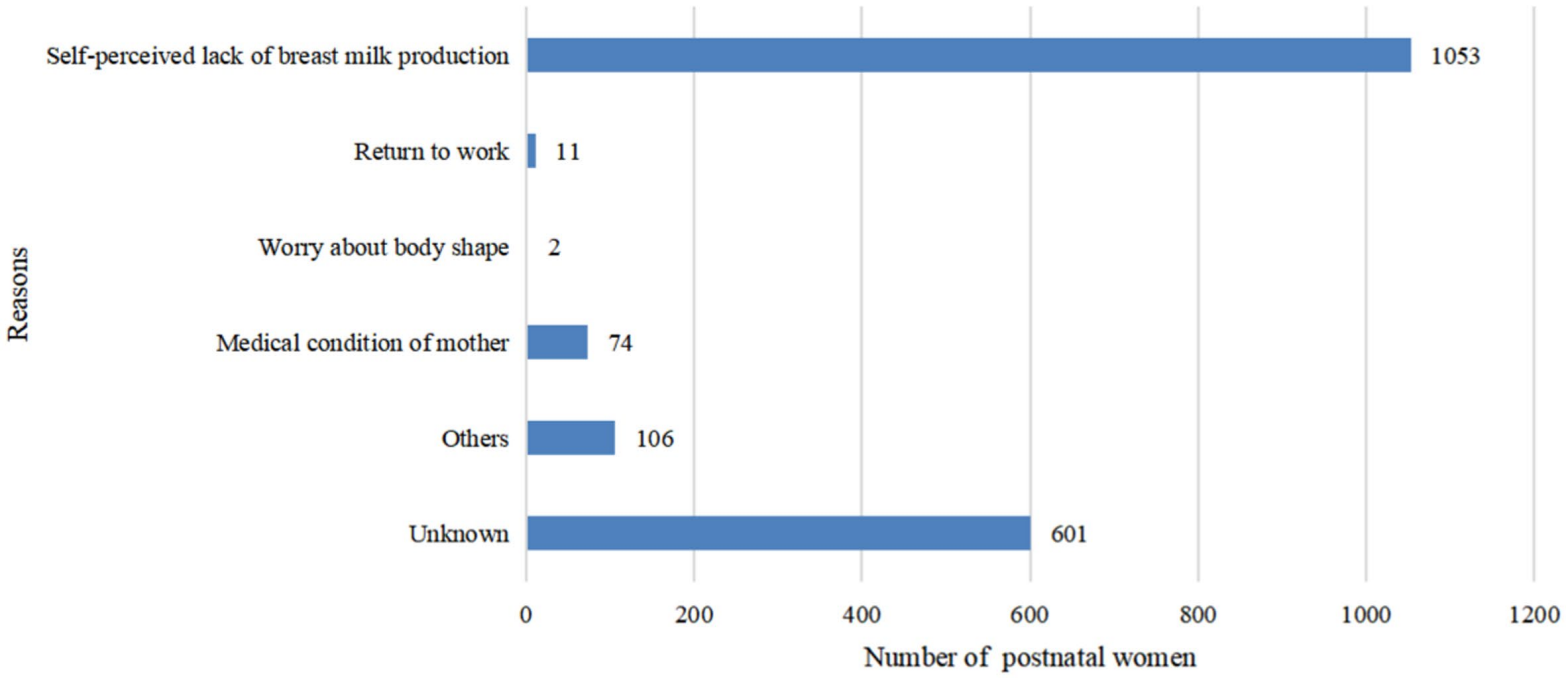
Reasons for non-exclusive breastfeeding within 42 days after delivery.

In Figure 1, we observed 32.54% (*n* = 601) of the participants did not give their answers. Among available date, we found the most frequent reasons given for breastfeeding cessation during puerperium were perceived low milk quantity (*n* = 1,053, 57.01%), medical condition in mother (*n* = 74, 4.01%) and others (*n* = 106, 5.74%).

### 3.3. Univariate analysis of influential factors of EBF

Univariate analyses of all variables (i.e., sociodemographic factors, BMI, delivery mode, parity, postnatal depression, breast or nipple abnormalities, premature birth, feeding knowledge score, and feeding attitude) that could possibly be associated with EBF at 42 days postpartum are shown in Table 2.

**Table 2:** Univariate analysis of influential factors of EBF.

Variables		NEBF	EBF	*t*/*χ*2	*p*-value
		*N* = 1847	*N* = 1891		
Age [mean (SD)]		30.42 (4.03)	30.07 (3.61)	2.85	0.004**
BMI		21.34 (3.08)	21.18 (2.88)	1.67	0.096
Feeding knowledge score		9.99 (2.83)	10.23 (2.78)	−2.60	0.009**
Nationality (%)	Han	1,690 (91.5)	1776 (93.9)	8.10	0.004**
	Minority	157 (8.5)	115 (6.1)		
Residence (%)	Urban	1,662 (90)	1736 (91.8)	3.74	0.053
	Rural	185 (10)	155 (8.2)		
Educational level (%)	Low	226 (12.2)	191 (10.1)	7.57	0.023*
	Median	126 (6.8)	105 (5.6)		
	High	1,495 (80.9)	1,595 (84.3)		
Delivery (%)	Natural delivery	1,171 (63.4)	1,319 (69.8)	16.95	<0.001***
	Cesarean section	676 (36.6)	572 (30.2)		
Parity (%)	Primipara	1,306 (70.7)	1,286 (68)	3.21	0.073
	Multipara	541 (29.3)	605 (32)		
Breast or nipple abnormalities (%)	No	1,693 (91.7)	1719 (90.9)	0.67	0.412
	Yes	154 (8.3)	172 (9.1)		
PPD (%)	No	1,333 (72.2)	1,407 (74.4)	2.38	0.123
	Yes	514 (27.8)	484 (25.6)		
Premature birth (%)	No	1723 (93.3)	1793 (94.8)	3.92	0.048*
	Yes	124 (6.7)	98(5.2)		
Feeding attitude (%)	Neutral or unwilling	142 (7.7)	22 (1.2)	94.83	<0.001***
	Willing	1705 (92.3)	1869 (98.8)		
Income (%)	Low	492 (26.6)	475 (24.5)	1.23	0.542
	Median	653 (35.4)	691 (38)		
	High	702 (38)	725 (37.5)		

Chi-square test, independent *t*-test. *p < 0.05, **p < 0.01, ***p < 0.001. NEF, non-exclusive breast feeding (bottle feeding or mixed feeding); EF, exclusive breast feeding.

Factors related to the cessation of EBF at 42 days were higher maternal age (30.42 ± 4.03 vs. 30.07 ± 3.61 *p* = 0.004), minority groups (8.5% vs. 6.1% *p* = 0.004), having lower educational level (12.2% vs. 10.1% *p* = 0.023), underwent cesarean (36.6% vs. 30.2% *p* < 0.001), preterm birth (6.7% vs. 5.3% *p* = 0.048), lower feeding knowledge score (9.99 ± 2.83 vs. 10.23 ± 2.78, *p* = 0.009), subjectively feel unwilling or neutral to adopt EBF (7.7% vs. 1.2% *p* < 0.001).

Besides, in univariate analysis, we found no significant differences in parity, family monthly income, breast or nipple abnormalities and the prevalence of PPD.

### 3.4. Bivariate logistic regression analysis of influential factors of EBF after delivery

Bivariate logistic regression analysis was conducted to investigate the potential associated factors of EBF in Table 3. Feeding mode (EBF or NEBF) during the 42 days after childbirth was settled as outcome variable. Variables showed association in bivariate analysis at *p*-value ≤0.10 were taken into the model to control confounders. These variables were age, nationality, residence, BMI, educational level, delivery mode, parity, premature birth, feeding attitude, and feeding knowledge score.

**Table 3:** Bivariate logistic regression analysis of influential factors of EBF within 42 d after delivery.

Variables		*b*	SE	Wald χ2	OR	95%CI	*p*-value
Age		−0.045	0.01	20.91	0.96	0.94–0.98	<0.001***
Feeding knowledge score		0.08	0.11	45.701	1.08	1.06–1.11	<0.001***
Nationality	Han						
	Minority	−0.38	0.13	8.73	0.68	0.53–0.88	0.003**
Delivery	Natural vaginal delivery						
	Cesarean section	−0.27	0.07	14.36	0.76	0.66–0.88	<0.001***
Parity	Primipara						
	Multipara	0.28	0.08	11.87	1.32	1.13–1.55	0.001**
Feeding attitude	Neutral or unwilling						
	Willing	1.90	0.23	66.23	6.69	4.23–10.57	<0.001***

*p < 0.05; **p < 0.01; ***p < 0.001. NEF, non-exclusive breast feeding (bottle feeding or mixed feeding); EF, exclusive breast feeding. Adjusted for residence, BMI, educational level, and premature birth.

Factors related to EBF at 42 days postpartum in regression analysis were as follow: Every single year increased in age, 4% less likely to adopt EBF (OR = 0.96, 95%CI 0.94–0.98, *p* < 0.001). Compared with Han, Ethnic minority women were 32% less likely to breastfeed exclusively (OR = 0.68, 95%CI 0.53–0.88, *p* = 0.003). Mothers went through cesarean section were 24% less likely to adopt EBF in relation to natural vaginal delivery (OR = 0.76, 95%CI 0.66–0.88, *p* < 0.001). And multiparas were 32% more likely to breastfeed exclusively than primiparas (OR = 1.32, 95%CI 1.13–1.55, *p* = 0.001). Women who were subjectively willing to breastfeeding were significantly more likely to adopt EBF (6.69, 95%CI 4.23–10.57, *p* < 0.001). Aside from this, every single point rise in feeding knowledge score, 8% more likely for mothers to adopt EBF (OR = 1.08, 95%CI 1.06–1.11, *p* < 0.001).

### 3.5. Subgroup analysis of feeding knowledge score and EBF

To investigate the association between feeding knowledge score and exclusive breastfeeding, we conduct subgroup analysis by age (< 35 vs. ≥ 35 years), nationality, delivery mode, parity and feeding attitude. Each analysis adjusted for variables with *p* < 0.1 in univariate analysis.

**Figure 2:**
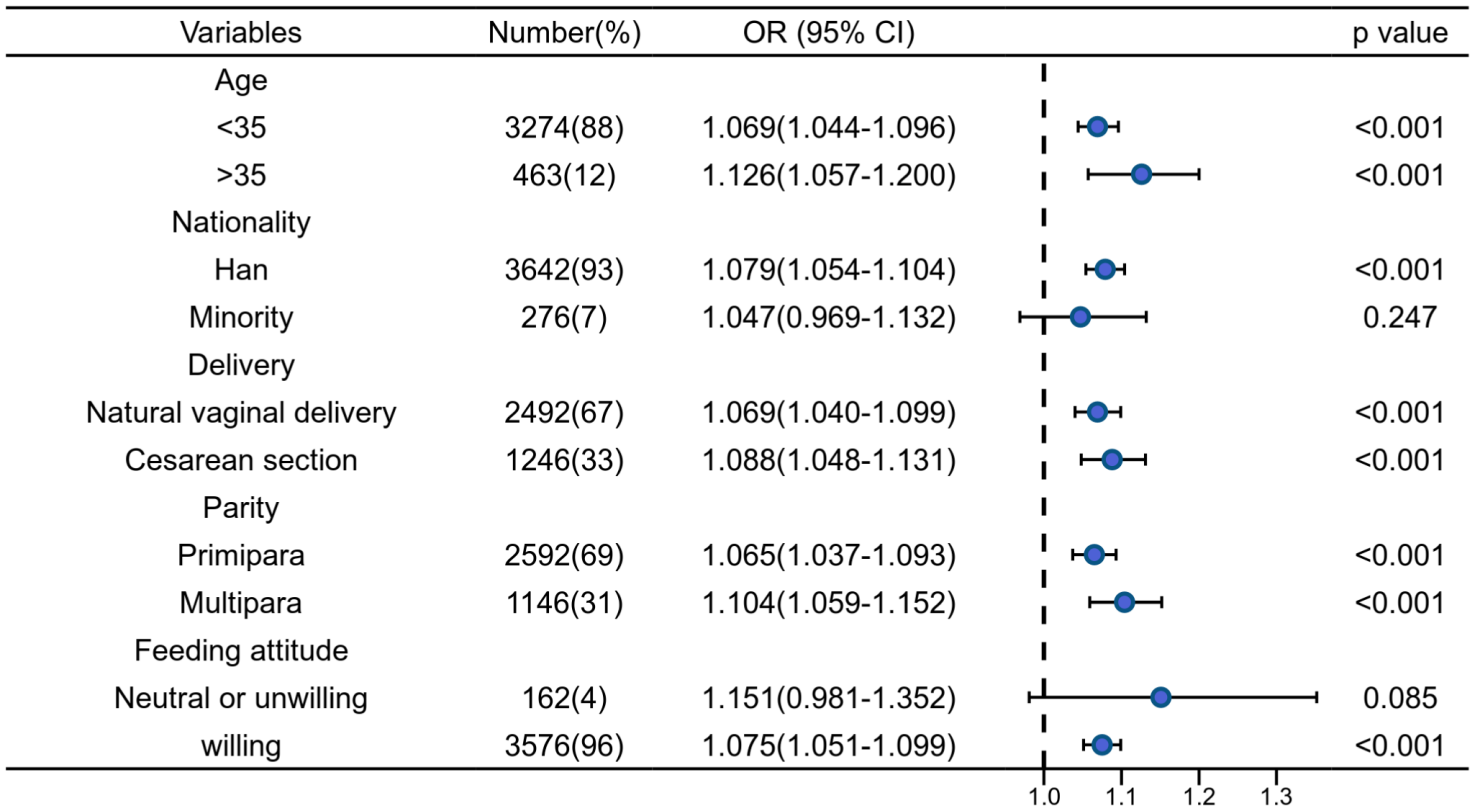
Bivariate logistic regression analysis of association between the feeding knowledge score and EBF stratified by selected factors.

According to Figure 2 we can find that the results of the subgroup analysis are relatively robust. Except for ethnic minorities and those with less positive attitudes towards breastfeeding, breastfeeding knowledge scores had a positive effect on mothers’ exclusive breastfeeding behavior (*p* < 0.05). If the breastfeeding knowledge score get improved, the rates of EBF may follow suit (OR > 1).

### 3.6. Correct rate of breastfeeding knowledge

**Figure 3:**
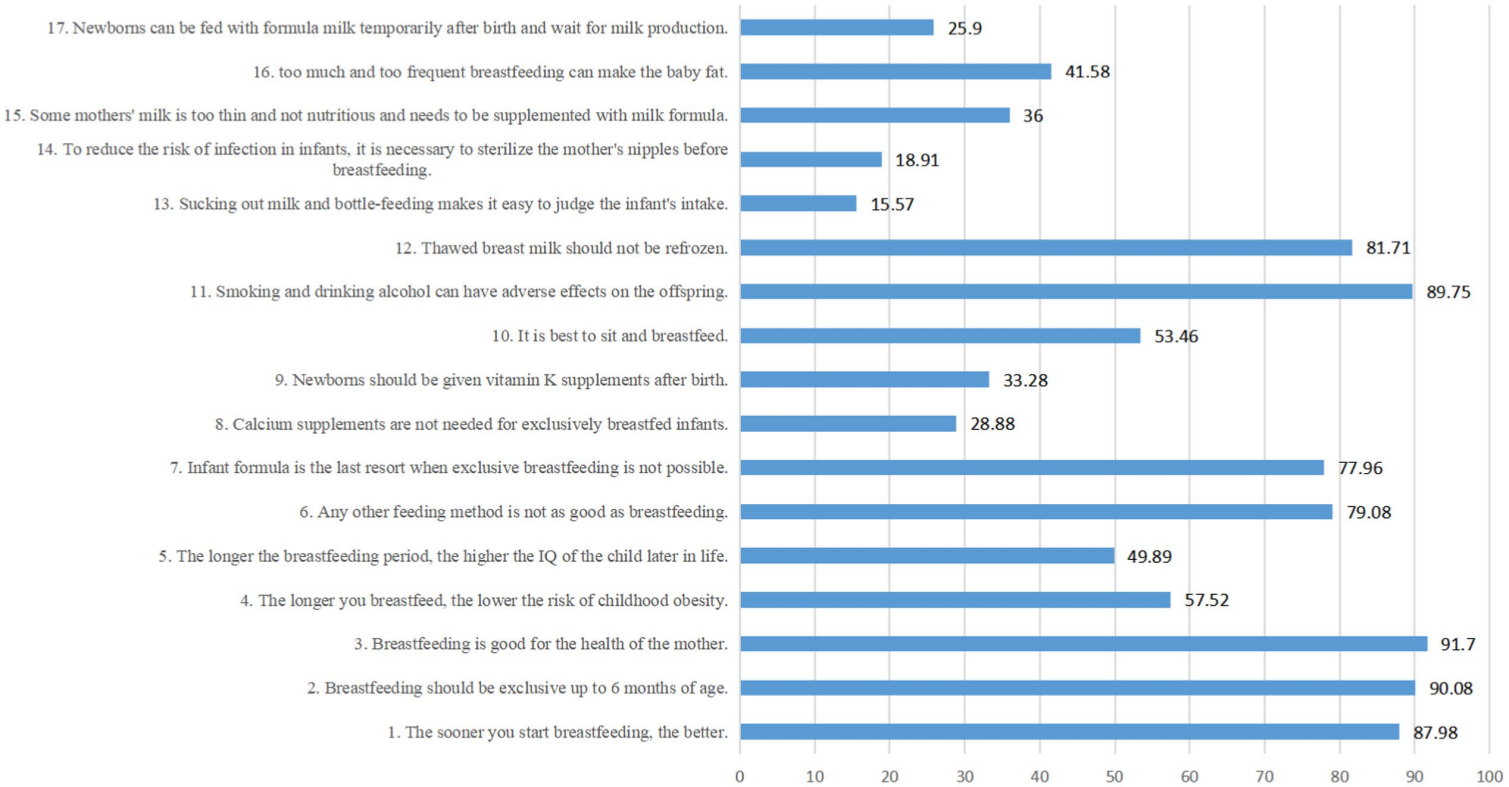
Correct rate of breastfeeding knowledge.

We illustrated the correct rate of each item of breastfeeding knowledge in Figure 3. For the following basic knowledge, the overall correct rates of all mothers are above 80%, such as:

1. The sooner you start breastfeeding, the better. (√)
2. Breastfeeding should be exclusive up to 6 months of age. (√)
3. Breastfeeding is good for the health of the mother. (√)
11. Smoking and drinking alcohol can have adverse effects on the offspring. (√)
12. Thawed breast milk should not be refrozen. (√)

However, the mothers had poor knowledge about the following items, with overall correct rates less than 30%:

8. Calcium supplements are not needed for exclusively breastfed infants. (√)
13. Sucking out milk and bottle-feeding makes it easy to judge the infant’s intake. (×)
14. To reduce the risk of infection in infants, it is necessary to sterilize the mother’s nipples before breastfeeding. (×)
17. Newborns can be fed with formula milk temporarily after birth and wait for milk production. (×)

## 4. Discussion

The 42nd day after delivery is a critical point for mothers and health care workers. Firstly, a great majority of the postnatal women would come back to the hospital at this time, which provides doctors opportunity to talk to them face to face. With a high probability, the doctors are whom mothers have already met during pregnancy, so mothers would feel a sense a familiarity and trust. Therefore, this kind of interfere would be more effective than any other sources. Secondly, those who plan to give up EBF due to exhaustion or other reasons can be found at a relatively early time. With professional support and suggestions, it would be conducive for mothers to continue breastfeeding. Therefore, timely detection of mothers’ feeding difficulties contributes to timely interventions, benefiting EBF practice.

To the best of our knowledge, this is the first study to investigate the associated factors for EBF at the 42nd day postpartum in China. According to a survey conducted in Japan, more than 90% of pregnant women hope to breastfeed their children. Nevertheless, only 50% of mothers are able to continue EBF with their children up to 3 months postpartum (12). We had similar findings, our study showed that the overwhelming majority of mothers were willing to breastfeed (*n* = 3,574 95.6%), but only 50.6% were able to adopt EBF up to 42 days postpartum.

Although this study is not completely nationally representative, the results do indicate that the EBFR in China needs to be improved. And we have some main advantages. First, we adopted stricter inclusion criteria, only mothers gave birth to a single child this time were included, the influence of double or multiple births was excluded. Second, a subgroup analysis of the effect of breastfeeding knowledge score and EBF was carried out, proving the robustness and making the range of benefited population more precise. Moreover, the variables (psychological factor and BMI) that had failed to be considered in previous studies (22, 23) were taken into account by the present study.

### 4.1. Factors associated with lower EBFR

#### 4.1.1. Cesarean section

It is consistent with previous studies which showed lower EBFR after cesarean delivery than after vaginal delivery (14). To a certain extent, the pain after cesarean section can affect patients’ sleep quality and bring limitation to mothers’ position. On the other hand, recovery would take a longer time, together with other negative effects such as dietary restrictions, pain of the incision, and anxiety etc., leading to adverse impact on the secretion of milk. A study in Japan showed that anesthesia during cesarean delivery is not conducive to EBF (12). Therefore, early essential care needs to be improved for C-section mothers and newborns in China.

#### 4.1.2. Higher maternal age

According to some previous researches and the present investigation, higher maternal age is associated with lower EBFR (12, 22). Young women are more likely to EBF with their child, probably because they have more access to breastfeeding knowledge, while middle-aged and older adult women are more likely to have complications during pregnancy and postpartum. However, there was also a study found no relationship between maternal age and EBF (24). Moreover, a study in Brazil showed that mother under the age of 20 tend to introduce complementary food earlier in baby’s life. Therefore, adolescent mothers were less likely to EBF with their children (25). And a 2017 study in China suggested that older women were more likely to have a second child, with more feeding skills and experience, generating higher rates of EBF and longer duration (26).

#### 4.1.3. Premature birth

Furthermore, a study conducted by Inano et al. (12) has shown that full-term babies are more likely to be breasted (12). Preterm infants may need to be transferred to the pediatric department for observation and treated with an incubator. Long time of separation for mother and infant results in the absence of sucking stimulation, leading to the delay of lactation. Therefore, some mothers may suffer from mastitis due to milk retention, increasing the difficulty of EBF further. In our study, univariate analysis showed an association between gestational age at birth and EBF, but multivariate adjusted analysis found no statistical association.

#### 4.1.4. PPD

Current researches come to an agreement in term of the negative impacts of PPD on EBF, studies believe that mothers suffering from postpartum depression may delay the onset time of lactation, resulting in the reduction of milk secretion, and would have negative impacts on EBF and the duration (27). However, our study found no significant difference of the PPD incidence between EBF and NEBF groups, and the proportion of EBF was even slightly higher among women having PPD. Firstly, it might be that respondents sometime were unwilling to give us their true thoughts due to social expectations or mental stress, leading to inaccurate results. Secondly, what we performed was a correlation analysis and did not indicate causality, so the results need to be treated with caution.

A Canadian study showed that the score of EPDS (Edinburgh Postnatal Depression Scale) ≥10 is a predictor of low EBFR (28). Surveys conducted by different teams subsequently with large sample data showed that PPD could reduce EBFR, and the differences were significant (29–31). However, whether EBF would reduce the incidence of PPD remains unclear. Some studies answered in the affirmative. A multicenter survey conducted by Hatton et al. found that breastfeeding mothers had fewer depressive symptoms (32). A 2016 Malaysian prospective cohort study concluded that EBF was negatively associated with PPD (33). In 2017, Korean researchers published a survey with data derived from 81,447 women during 2002 to 2013. Compared with women who continued breastfeeding, those who stopped within 12 weeks after childbirth had a significantly higher incidence of PPD (34). However, there were also some studies showed no correlation between breastfeeding and PPD. A 2015 prospective cohort study conducted by Sukhee Ahn in Korea showed no significant difference in the incidence of PPD among 119 postnatal women from 7 days to 6 months postpartum (35). In a 2016 survey by Carley J.P, the regression analysis showed no association between breastfeeding duration and PPD at 5 to 7 months postpartum (36).

At present, researchers at home and abroad have agreed on the adverse impacts of PPD on EBF, but if breastfeeding can help prevent PPD remains controversial. More surveys are needed to determine whether women who stop breastfeeding early have a higher risk of PPD, and whether lower EBFR is associated with higher rates of PPD.

#### 4.1.5. Insufficient milk

Moreover, insufficient milk is an important reason for breastfeeding cessation in the present study, which is consistent with the results of previous studies (16, 37, 38). Plenty of studies showed the important role of nutritional factors played in EBF, and problems with low milk quantity usually occur within the first 2 weeks postpartum (39). In a study by Shi et al., nearly two-thirds of mothers stated they stopped EBF before 6 months postpartum because they did not think they had enough milk (23). Lewallen and colleagues found that around 30% of the mothers stopped EBF before 8 weeks postpartum, and the most common reason was the perception of insufficient milk supply. Therefore, they are prone to stop EBF and choose formula supplementation (37). Clinicians can carry out interventions, such as informing mothers the normal frequency of breastfeeding, and the flexibility of time and amount, which may be helpful to improve EBFR.

### 4.2. Factors associated with higher EBFR

#### 4.2.1. Higher educational level

Univariate analysis of this study showed the association between educational level and EBF, which is consistent with other studies (22, 40), but multivariate adjusted analysis found no statistical association.

Well educated women may obtain more breastfeeding knowledge and are more likely to insist on what they think is right. Moreover, women with higher educational level may have better control over their daily life as well as the work environment, contributing to longer periods of breastfeeding. However, a study in China (41) showed the EBFR is lower among women with higher educational level. It may be that well-educated women are more likely to afford formula, and higher educational level women are employed and need to return to work earlier after maternity leave, with no enough time and no suitable places for breastfeeding.

#### 4.2.2. Multipara

Univariate analysis in our study showed no association between parity and EBF, but multivariate adjusted analysis found multiparas were significantly more likely to EBF. In a previous study (42), they also found lower breastfeeding rates in primiparas due to inadequate parenting experience. However, results on breastfeeding and parity varied from study to study. A study conducted in China showed that women gave birth more than once do not have better EBFR than primiparas due to higher pressure of caring for more than one child, even though they had more parenting experience (26).

#### 4.2.3. High breastfeeding knowledge score and positive feeding attitude

According to the TPB (Theory of Planned Behavior), knowledge was the largest factor associated with behavior, followed by subjective norms, practice control, and attitudes (43). Our study also found that the higher the maternal breastfeeding knowledge score was, the more likely mothers were to breastfeed their infants exclusively within 42 days postpartum. This is also illustrated in a research by Ishak et al., which showed that having a higher level of education and good perception towards breastfeeding was more likely to result in the mothers being highly motivated and confident to exclusively breastfeed (44). Shafaei et al. also stated in their study that providing mothers, particularly those who previously had problems in breastfeeding, with counseling in healthcare centers or through online media during postpartum period, might help to improve EBFR (45). Furthermore, the breastfeeding knowledge informed by medical staffs would produce a higher degree of trust and generate better compliance among mothers. Thus, health care workers should reinforce the education about breastfeeding during pregnancy, hospitalization and rechecks, timely help postnatal women solve the difficulties encountered in breastfeeding.

In subgroup analysis, there was no significant association between breastfeeding knowledge score and EBF among ethnic minorities and people with negative attitudes towards breastfeeding.

Ethnic minorities (*n* = 276, 7%) and feel neutral or unwilling to breastfeed (*n* = 162, 4%) are groups with a small number of people, it is difficult to draw significant results. Additionally, many women are less positive to breastfeeding because they would feel embarrassed and humiliated when families, friends and doctors watch them breastfeed. Therefore, for mothers with a negative attitude, improving breastfeeding knowledge may not be the optimal solution.

### 4.3. Limitations of the study

Some limitations must be taken into account when interpreting these results. First, this was a single center retrospective study conducted in Northwest China, and only evaluated breastfeeding status up to 42 days postpartum. Therefore, our results may lack of national representative and cannot be compared to other studies with longer periods of breastfeeding duration. Second, this study covered potential associated factors of EBF among multiple levels, but some variables in relation to environmental management and policy had failed to be measured. Third, the reasons for EBF cessation were based on mother’s subjective judgements and some others reason have not been considered, such as “mother/infant separation; breastfeeding skills were not effective; inconvenience/fatigue due to breastfeeding; baby’s medical condition” (39, 46). Finally, respondents tend to choose answers that meet social expectations rather than their true thoughts and behaviors, which may lead to inaccurate results. Thus, social desirability bias should also be taken into consideration.

### 4.4. Future outlook

Perceiving insufficient breast milk of mothers is the most common reason for stopping EBF. Therefore, difficulties encountered in the process of breastfeeding should be solved in a timely manner, mothers’ confidence in breastfeeding can be improved by offering them more knowledge and counseling. Aside from this, for those of ethnic minority or those refused to breastfeed, more researches are needed to explore the uncovered reasons, therefore more personalized interventions could be made.

We need future studies to continuously monitor the trend of EBFR, explore effective interventions and develop a comprehensive and individualized framework of strategies to support children, mothers and their families. And we devoutly suggest that health institutions should control the rates of cesarean section, and supportive environments for postpartum mothers to breastfeeding should be ensured in public and the workplace. Moreover, the government should provide support to improve EBFR, including raising the social and cultural acceptance of breastfeeding among public, providing special rooms for breastfeeding in public places, ensuring equity and welfare in women’s employment, and providing adequate paid maternity leave for mothers.

## 5. Conclusion

In this study, higher age, ethnic minorities and cesarean section were associated with the cessation of EBF. However, protective factors of EBF included multipara, positive feeding attitude and high breastfeeding knowledge score. According to the TPB (Theory of Planned Behavior), knowledge was the biggest factor associated with exclusive breastfeeding behavior (43). Therefore, we conducted a subgroup analysis to determine the target groups for future intervention. We found that the breastfeeding knowledge score had a significant impact on a majority of puerperium women, except for those of ethnic minority and of those with less active breastfeeding attitudes. For most mothers, we should focus on breastfeeding education and provide them with more related programs to popularize the breastfeeding related knowledge. However, for the other small population, among whom breastfeeding knowledge is of limited use, more researches are needed to uncover the underlying reasons so that individualized strategies can be developed for them.

Although the vast majority of mothers had strong motivation and intention to breastfeed, many factors (sociodemographic characteristics of mother and child, physical and mental health, socio-cultural and environmental factors) may still affect the successful implementation of postpartum EBF. Thus, we believe we need a comprehensive and individualized framework of strategies to help improve the EBFR in China.

## Data availability statement

The raw data supporting the conclusions of this article will be made available by the authors, without undue reservation.

## Ethics statement

The studies involving human participants were reviewed and approved by Ethics Committee of The First Affiliated Hospital of Chongqing Medical University with approval number 2018-131. The patients/participants provided their written informed consent to participate in this study.

## Author contributions

LW and YZ contributed to the study conception and design. Questionnaires were provided by WW, HJ, and FW. YZ and YC gave statistics support. LW provided clinical support. The first draft of the manuscript was written by YC. YC, YZ, WW, FW, HJ, and LW commented on previous versions of manuscript. All authors contributed to the article and approved the submitted version.

## Funding

This project was funded by Discipline Cultivation Fund of the First Affiliated Hospital of Chongqing Medical University and Chongqing Social Science Planning Project (2017YBSH057) and Joint Program of the Chongqing Science and Technology Bureau and the Chongqing Health Commission (2021MSXM215).

## Conflict of interest

The authors declare that the research was conducted in the absence of any commercial or financial relationships that could be construed as a potential conflict of interest.

## Publisher’s note

All claims expressed in this article are solely those of the authors and do not necessarily represent those of their affiliated organizations, or those of the publisher, the editors and the reviewers. Any product that may be evaluated in this article, or claim that may be made by its manufacturer, is not guaranteed or endorsed by the publisher.
