# Supplementary material for: Factors associated with exclusive breastfeeding during postpartum in Lanzhou city, China: a cross-sectional study
Source: Front Public Health. 2023 Aug 30;11:1089764. doi: 10.3389/fpubh.2023.1089764 (PMC10498539; doi:10.3389/fpubh.2023.1089764)
Supplement: Supplementary file 1 [file Data_Sheet_1.PDF]

Breastfeeding knowledge

1. The sooner you start breastfeeding, the better.
2. Breastfeeding should be exclusive up to 6 months of age.
3. Breastfeeding is good for the health of the mother.
4. The longer you breastfeed, the lower the risk of childhood obesity.
5. The longer the breastfeeding period, the higher the IQ of the child later in life.
6. Any other feeding method is not as good as breastfeeding.
7. Infant formula is the last resort when exclusive breastfeeding is not possible.
8. Calcium supplements are not needed for exclusively breastfed infants.
9. Newborns should be given vitamin K supplements after birth.
10. It is best to sit and breastfeed.
11. Smoking and drinking alcohol can have adverse effects on the offspring.
12. Thawed breast milk should not be refrozen.
13. Sucking out milk and bottle-feeding makes it easy to judge the infant's intake.
14. To reduce the risk of infection in infants, it is necessary to sterilize the mother's nipples before breastfeeding.
15. Some mothers' milk is too thin and not nutritious and needs to be supplemented with milk formula.
16. too much and too frequent breastfeeding can make the baby fat.
17. Newborns can be fed with formula milk temporarily after birth and wait for milk production.

Options: (1) yes (2) no (3) don't know

Answers:1-12: Yes; 13-17: No
